# Bacterial magnetosomes loaded with doxorubicin and transferrin improve targeted therapy of hepatocellular carcinoma

**DOI:** 10.7150/ntno.34601

**Published:** 2019-07-08

**Authors:** Jiaojiao Wang, Yuanyuan Geng, Yunpeng Zhang, Xi Wang, Junquan Liu, Abdul Basit, Ting Miao, Weiquan Liu, Wei Jiang

**Affiliations:** Beijing Advanced Innovation Center for Food Nutrition and Human Health, State Key Laboratory of Agro-Biotechnology, College of Biological Sciences, China Agricultural University, Beijing 100193, China.

**Keywords:** Bacterial magnetosomes, doxorubicin, transferrin, antitumor effect, hepatocellular carcinoma

## Abstract

High metastatic rate and recurrence of tumor because of tumor circulating cells are seriously hinders for clinical tumor therapy. Herein, we develop a novel, active**-**targeting nanotherapeutic by simultaneously loading doxorubicin (DOX) and transferrin (Tf) onto bacterial magnetosomes (Tf-BMs-DOX) and investigate its antitumor efficacy *in vitro* and *in vivo*. Drug release profiles indicated that Tf-BMs/BMs loaded with DOX were capable of sustained drug release, suggesting that reduce drugs required frequency of administration and enhance their therapeutic effect. The results of cellular uptake revealed that Tf-BMs-DOX recognized hepatocellular carcinoma HepG2 cells more specifically compared to HL-7702 normal hepatocytes because of high expression of transferrin receptor (TfR) on the surface of HepG2 cells. Tf-BMs-DOX increased tumor cytotoxicity and apoptosis more significantly than free DOX or BMs-DOX by regulating the expression of tumor**-**related and apoptosis**-**related genes. Following intravenous injection in HepG2 cell**-**bearing mice, Tf-BMs-DOX displayed tumor suppression rate of 56.78%, significantly higher than that of the BMs-DOX (41.53%) and free DOX (31.26%) groups. These results suggest that Tf-BMs-DOX have the potential to actively target to tumor sites, as well as the ability to kill circulating tumor cells via intravenous injection. Our findings provide a promising candidate for the clinical treatment of metastatic cancer.

## Introduction

In recent years, targeted nanotherapeutics for cancer has developed rapidly 1-3. The small size, diverse composition, surface functionalization, and good stability of targeted nanoparticles make them attractive for biomedical applications 4-6. Drug-loaded nanoparticles modified with specific ligands can bind to receptors on the surface of tumor cells, leading to the accumulation of nanoparticles on the surface of certain cells 7, 8. The specific interaction of ligands and their receptors allows nanoparticles target to specific cell types, which reduces the damage to normal cells. Since these ligand**-**receptor systems are non**-**toxic, non**-**immunogenic and biodegradable 9, the modification of nanoparticles with specific ligands has become a rapidly growing field in cancer therapy 10, 11.

Iron is a required factor that is involved in a variety of cellular processes including metabolism and DNA synthesis 9, 12. The transport of iron between its absorption, storage, and utilization sites is mediated by transferrin (Tf), an iron binding protein 9. Transferrin receptor (TfR), also known as CD71, is a type II transmembrane glycoprotein (180 kDa) and has an extracellular structure that binds to Tf 12-14. TfR exists in all nucleated cells and has low expression level in normal cells. However, its expression level in tumor cells is significantly increased, and is believed to be up to 100**-**fold higher in tumor cells compared to normal cells 12, 15-17. The high expression of TfR is found to be closely related to tumor stage 18. Therefore, TfR represents an attractive candidate to target tumor cells, since it is a relatively stable cell surface antigen.

Doxorubicin (DOX), an antibiotic anthracycline chemotherapeutic agent, has been used as an effective therapeutic drug in many types of tumor 19**.** Its antitumor activity arises after it interacts with DNA, after which it inhibits the activity of topoisomerase and generates free radicals. Despite its high antitumor efficiency, the application of DOX is severely hampered by its adverse effects to healthy tissues, notably its cardiotoxicity 20-22. Many strategies have been attempted to improve the therapeutic efficacy of DOX while reducing its toxic effects, among which the application of nanodrug delivery system shows great advantages.

Bacterial magnetosomes (BMs) coated by organic membranes are a promising new type of magnetic nanoparticle 23, 24. BMs have a uniform particle size (40**-**50 nm), strong magnetism, and a membrane coated with chemical groups such as **-**NH_2_,** -**COOH,** -**OH, which makes them easy to conjugate with antibodies, genes, and drugs. Due to these properties, BMs have become promising candidates for biomedical and biotechnology applications, especially in cancer therapy 25-34. Great progress has been made in the using BMs for applications such as antibody and drug coupling technology 35. In previous studies, we prepared DOX**-**loaded BMs performed subcutaneous injection of BMs-DOX adjacent to tumor sites. We found that drug-loaded BMs exhibit significant lethality on human hepatocellular carcinoma cells, with reduced toxicity to normal cells 36, 37.

Due to the high metastatic rate of tumors, the recurrence rate of hepatocellular carcinoma is as high as 60-70% after treatment. One of the key factors underlying recurrence and metastasis of cancer after a tumorectomy is the prevalence of circulating tumor cells (CTCs) in the peripheral blood 38, 39. The current markers used to detect CTCs include phosphatidylinositol proteoglycan-3 40, 41, transferrin receptor 42, Golgi protein 73 43, alpha fetoprotein 44, α-L-fucosidase 45, and epithelial cell adhesion molecule 46-48, among others. Nanoparticles targeted to markers of CTCs can inhibit the growth of tumors 40-42 and thus greatly reduce the metastasis and recurrence of tumors after surgery.

**Scheme 1:**
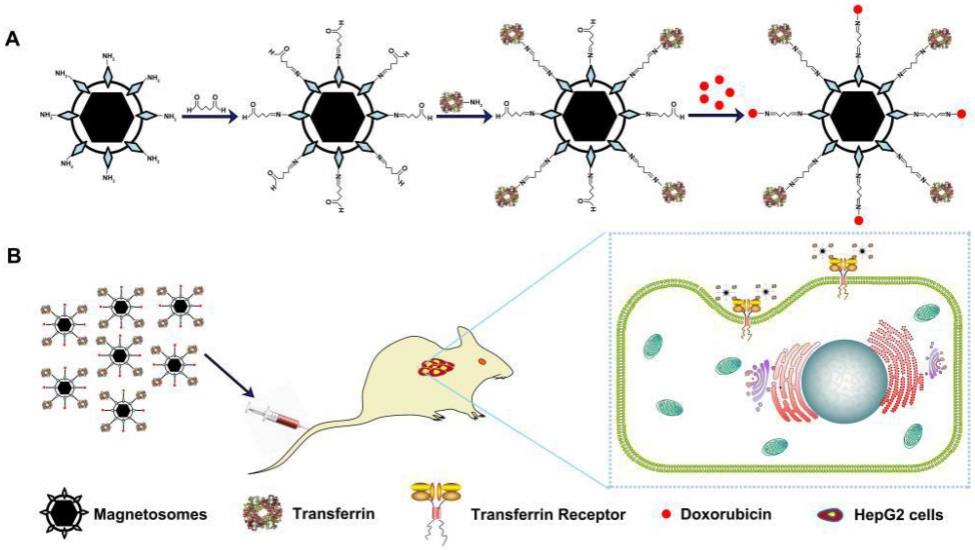
** (A)** Schematic depiction of formulation of Tf-BMs-DOX. **(B)** The anti-tumor of Tf-BMs-DOX *in vivo*.

In this study, we construct actively target drug-loaded nanoparticles (loaded DOX and transferrin on to BMs, termed Tf-BMs-DOX), which recognized tumor cells by interaction with Tf and TfR on tumor cells, and examine the therapeutic efficacy of Tf-BMs-DOX *in vitro* and *in vivo* (Scheme 1). Our results revealed that Tf-BMs-DOX target hepatocellular carcinoma HepG2 cells more effectively than the normal hepatocyte HL-7702 line. Tf-BMs-DOX increased tumor cell cytotoxicity and apoptosis more significantly than free DOX or BMs-DOX by regulating the expression of tumor**-**related and apoptosis**-**related genes. To examine the targeting effect of Tf-BMs-DOX, all the BMs reagents were administered by intravenous injection to tumor bearing mice. Compared to BMs without Tf loaded on, Tf-BMs-DOX showed a higher inhibition of tumor growth, with no side effects on normal tissues. In summary, this strategy may provide an effective method for targeted tumor treatment in clinics, and Tf-BMs-DOX may play an important role in inhibiting tumor metastasis and recurrence after tumor resection.

## Materials and methods

BMs were extracted from the *Magnetospirillum gryphiswaldense* MSR-1, was maintained in our laboratory. Holo-transferrin (Sigma Co., Ltd. USA), human hepatoma cell line HepG2 and human normal hepatic cell line HL-7702 were maintained in our laboratory. DOX hydrochloride was purchased from EDQM (Strasbourg, France), MTT, 4', 6'-diamidino-2-phenylindole dihydrochloride (DAPI) and Hoechst 33342 were from Solarbio Science & Technology (Beijing, Chin) Male BALB/c nude mice (aged 6-8 weeks) were obtained from Beijing Vital River Laboratory Animal Technology (Beijing, China).

### Preparation of Tf-BMs-DOX

Transferrin-functionalized DOX-loaded BMs was prepared using glutaraldehyde as a cross linker, to immobilize transferrin moieties on the BMs surface. Briefly, to remove extraneous proteins, BMs were treated with proteinase K and electro-eluted. BMs (1 mg) were rinsed by PBS (2 mL) and then modified with 25% of glutaraldehyde solution (with the final concentration of 5%, v/v). The solution was ultrasonicated for 1 minute followed by the intervals of 4 minutes (this step was repeated for 12 times). Transferrin solution (500 µg) was suspended with modified BMs and incubated at 37^o^C**,** 100 rpm for 1 h. The adsorbed magnetite was rinsed with PBS and DOX (500 µg) solution was suspended with transferrin-functionalized BMs. Tf-BMs-DOX were retrieved by magnetite adsorption followed by the incubation at 37^o^C, 100 rpm. The drug loading and encapsulation efficiency was calculated according to the following formula:

Drug loading (%) = Amount of drug loaded in BMs/Total amount of BMs × 100%

Encapsulation efficiency (%) = Amount of drug loaded in BMs/Total amount of drug added during fabrication × 100%

### Characterization of Tf-BMs-DOX

The Tf-BMs-DOX was characterized by transmission electron microscopy (TEM), Fourier transform infrared spectroscopy (FT-IR) and Zeta PALS analysis. TEM (JEM -1230, JEM; Japan) was used for the morphological study of BMs, BMs-DOX and Tf-BMs-DOX. FT-IR spectra were recorded by Nicolet iS50 NIR spectrometer (Thermo, USA). Zeta potential and hydrodynamic size for the measurements of BMs, BMs-DOX and Tf-BMs-DOX, were performed by Zeta PALS analysis (Brookhaven, USA).

### Cell Culture

HepG2 cells and HL-7702 cells were cultured and maintained in Dulbecco's Modified Eagle Medium (DMEM) with 10% (v/v) FBS (Gibco, America) and penicillin/streptomycin (1%) at 37^ o^C with 5% CO_2_/95% air atmosphere incubator.

### *In vitro* drug release

*In vitro* release of DOX from DOX-loaded BMs was investigated using dialysis method 11. Equal amount of DOX-loaded BMs (400 µg) and free DOX were dispersed into PBS (1.5 mL) and then loaded into the dialysis bags. Bags were placed in flasks containing 50 mL PBS (pH 7.4) and dialyzed in a reciprocating water bath shaker at 37^o^C, 100 rpm. Dialysis solution (4 mL) was extracted at various time points and replaced with the same volume of PBS. The concentration of DOX was quantified by high performance liquid chromatography (HPLC).

### TfR detection

The expression of TfR on the surface of HepG2 cells and HL-7702 cells were examined by confocal laser scanning microscope (CLSM) and flow cytometry. CLSM images were obtained by fixation of HepG2 and HL-7702 cells using paraformaldehyde (4%) for 15 min at room temperature. Cells were then incubated with 5% bovine serum albumin (BSA) and 0.1% Tween for 1 h, followed by incubation with rabbit anti-TfR antibody (1/500, v/v, in 0.01 M PBS, Abcam, MA, USA) at 4^o^C overnight. Cells were incubated with Alexa594-conjugated mouse anti-rabbit IgG (1/1000, v/v, in 0.01 M PBS Solarbio, China) for 1 h in darkness, and then stained with 100 µg/mL DAPI in darkness. The control group was incubated with 5% BSA to eliminate the nonspecific binding.

For flow cytometry, HepG2 and HL-7702 cells seeded in 6-well plates were suspended in PBS (at the concentration of 1×10^6^) and incubated with PE-conjugated anti-TfR (3 µL) for 30 min in darkness. Flow cytometry (FACS Calibur, BD Biosciences; San Jose, CA, USA) was performed to determine the expression of TfR on the surface of HepG2 cells and HL-7702 cells.

### Cellular uptake of Tf-BMs-DOX

Cellular uptake of Tf-BMs-DOX in HepG2 and HL-7702 cells were determined by CLSM and flow cytometry. HepG2 cells were seeded into 24-well plate containing coverslips (at the density of 5×10^4^ cells/well) and incubated for 12 h to reach 80% confluence at 37^o^C. Cells were incubated in fresh medium containing 10 µg/mL of free DOX, BMs-DOX and Tf-BMs-DOX for 1 h. Cell fixation were performed as described above and then stained with 100 µg/mL DAPI for 15 min at 37^o^C in the darkness. CLSM (model A1, Nikon, Japan) was performed for the qualitatively observation for the uptake of Tf-BMs-DOX in HepG2 cells. Quantitative measurement of Tf-BMs-DOX uptake by HepG2 cells were evaluated using flow cytometry. Cells were seeded into 12-well plate at the density of 1×10^5^ cells/well and incubated for 12 h to reach 80% confluence at 37^o^C, then treated with the fresh medium that containing 10 µg/mL of free DOX, BMs-DOX and Tf-BMs-DOX for various time points (1, 2, 4, 8 h). For each sample, 20,000 individual cells were collected for quantification.

### Intracellular localization of Tf-BMs-DOX

HepG2 cells were seeded into 24-well plate that containing cover slips at the density of 5×10^4^ cells/well and incubated for 12 h at 37^o^C. Cells were treated with the fresh medium that containing 10 µg/mL of free DOX, BMs-DOX and Tf-BMs-DOX for 4 h. After that, cells fixation was performed as described above and then the cells were stained with 100 µg/mL DAPI in darkness after rinsed by PBS, finally were stained with Lysotracker® Red DND-99 (Solarbio, China) for 30 min in darkness. CLSM was performed to detect the intracellular location of the Tf-BMs-DOX.

### *In vitro* cytotoxicity assay

The cytotoxicity of DOX-loaded BMs to HepG2 and HL-7702 cells was determined by methyl thiazoly tetrazolium (MTT) assay. Cells were seeded into 96-well plates (at the density of 1×10^4^ cells/well) and incubated for 12 h to reach about 80% confluence. Cells were treated with the fresh medium that containing different concentration of free DOX, BMs, BMs-DOX and Tf-BMs-DOX for 12-48 h. Cell viability was measured using MTT kit (Solarbio Science & Technolog) and the absorbance of cells was detected at 490 nm with a microplate reader (Power WaveXS2, BioTek Instruments; Winooski, VT, USA). Each experiment was performed in triplicate.

### Apoptosis assay

Considering that the red fluorescence of DOX affect the detection of apoptosis by FACS, we decreased the concentration of DOX to 0.5 µg/mL to avoid the interference. HepG2 and HL-7702 cells were seeded into 24-well plates that containing cover slips (at the density of 5×10^4^ cells/well). Cells were incubated with 0.5 µg/mL of free DOX, BMs-DOX and Tf-BMs-DOX for 12 h and 24 h. Cells fixation was performed as described above and then the cells were stained with 100 µg/mL Hoechst 33342 (Solarbio, China) for 15 min at 37^o^C in the darkness. Fluorescent microscopy (AopTome, Germany) was used to detect the formation of apoptosis body in the cells.

Apoptosis was analyzed by Annexin V/PI double staining kit (Solarbio, China). Briefly, HepG2 cells seeded at 12-well plates were incubated with 0.5 µg/mL of free DOX, BMs-DOX and Tf-BMs-DOX for 24 h and 48 h. Cells were suspended in 500 µL 1×binding buffer after washing by cold PBS, and then 3 µL AnnexinV-FITC and 1 µL PI were added into each well and incubated for 15 min in the darkness. For each sample, 20,000 individual cells were collected for quantification.

### Quantitative real-time PCR

HepG2 and HL-7702 cells were seeded into 12-well plate (at the density of 1×10^5^ cells/well) and incubated for 12 h at 37^o^C. Cells were treated with the fresh medium containing 0.5 µg/mL of free DOX, BMs-DOX and Tf-BMs-DOX for 12, 24 and 48 h. RNA was isolated using Total RNA Purification Kit (Genemark, Taiwan) following the manufacturer's instructions. Quantitative real-time PCR was performed using Fast SYBR Mixture (with High ROX) (CWBIO, Beijing, China). All samples were performed in triplicate.

### *In vivo* anti-tumor of drug-loaded BMs

Male BALB/c nude mice were inoculated subcutaneously into right flank with HepG2 cells (0.1 mL of 1×10^7^ cells/mice). Tumor volume was calculated as A × B^2^/2, where A is length and B is width diameters (mm) of tumors. When average tumor volume exceeded 100 mm^3^, mice were divided into 5 groups (n=6) and injected intravenously with 4 mg/kg (DOX content) BMs-DOX, Tf-BMs-DOX and free DOX drugs at 3-day intervals. Control group was injected with PBS. Body weight and tumor volume were measured every 2 days. When average tumor volume of control group reached 1000 mm^3^, mice were sacrificed. Tumors and major organs (heart, liver) were excised for hematoxylin-eosin (HE) staining and further studies.

### Detection of iron ions in tumor

A certain amount of tumor was transferred to the Eppendorf tube, and 100 µL of nitric acid were added to each 100 mg tumor. When the tumors were completely digested, Eppendorf tubes were placed at 100^o^C for 3 h. Then 100 µL of each sample was diluted into 5 mL of ultra-pure water and filtered through 0.45 µm microporous membranes. Inductively coupled plasma optical emission spectrometry (ICP-OES) (model iCAP 6300, Thermo Fisher) was used to detect the iron content of each sample.

### Statistical analysis

Statistical analysis was performed using student's t-test for two groups, and one-way ANOVA for multiple groups. All data are given as mean ± standard deviation (S. D). Differences are considered statistically significant as *p < 0.05; **p < 0.01; ***p < 0.001.

## Results and discussion

### Characterization of Tf-BMs-DOX

**Figure 1:**
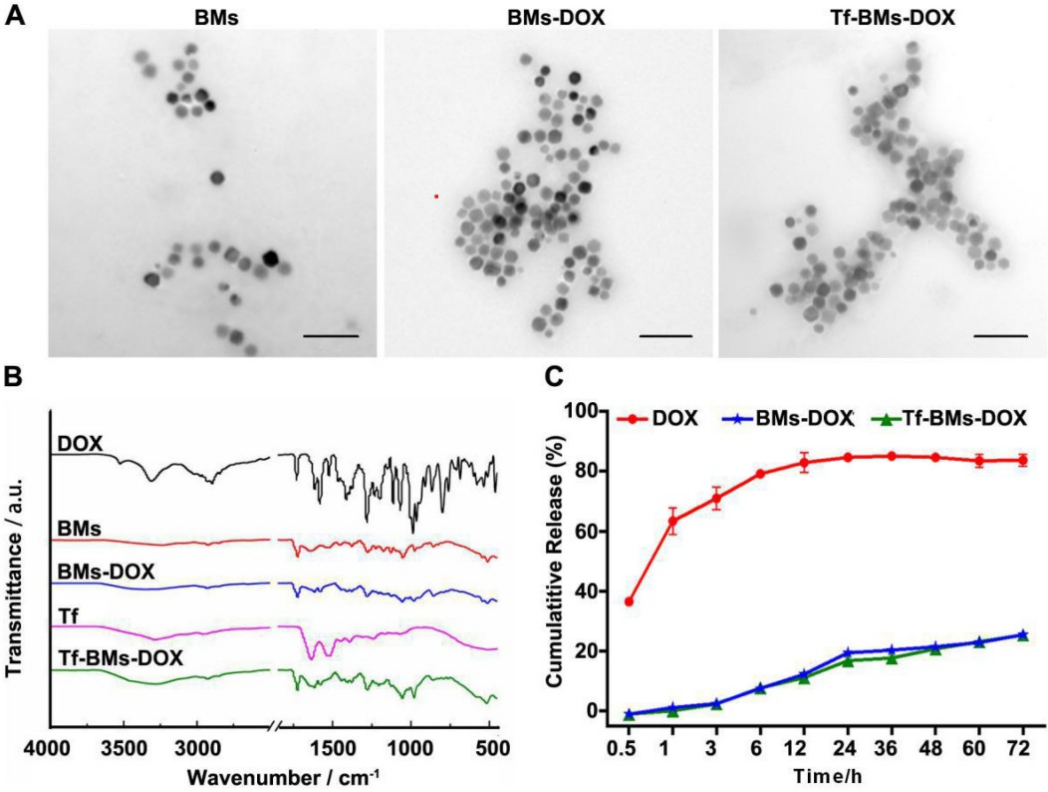
** Characterization of Tf-BMs-DOX. (A)** TEM images of BMs, BMs-DOX and Tf-BMs-DOX. Scale bar = 200 nm. **(B)** FT-IR spectrum of free DOX, free Tf, BMs, BMs-DOX and Tf-BMs-DOX. **(C)** DOX release profiles of free DOX, BMs-DOX and Tf-BMs-DOX.

In this study, BMs were chosen as the carrier for biofunctionalization on account of its high biocompatibility, high drug loading capacity, and versatile membrane surface groups. BMs-DOX or Tf-BMs-DOX nanoparticles were prepared via reaction between aldehyde and amino groups. We examined the morphology and size of non-loaded BMs, BMs-DOX, and Tf-BMs-DOX through transmission electron microscopy (TEM) and found no significant difference between the three BM types (Figure 1A). Analysis of size and zeta potential of Tf-BMs-DOX suspended in milli-Q water revealed that the average hydrodynamic sizes of BMs-DOX (630.50 nm) and Tf-BMs-DOX (702.86 nm) increased slightly compared with that of non-loaded BMs (418.02 nm) (Table 1). The loading of Tf and DOX to the surface of BMs reduced the Zeta potential of non-loaded BMs.

We performed Fourier-transform infrared (FT-IR) spectroscopy of Tf-BMs-DOX to analyze their surface groups. Compared with free DOX, the characteristic peak of Tf-BMs-DOX disappeared or weakened at 3525 cm^-1^, while the absorption peak of Tf-BMs-DOX was wide and scattered at 3280 cm^-1^. When comparing Tf-BMs-DOX to BMs, we found that Tf-BMs-DOX amide bond at 1616 cm^-1^, 1578 cm^-1^ were enhanced. This suggests that amide bonds formed during the reaction process, and the amide reaction may occur either on N-H or O-H. Compared with Tf, the absorption peak of amide Ⅲ band appeared at 1280 cm^-1^, indicating that transferrin was successfully coupled onto BMs (Figure 1B).

Drug release profiles of free DOX, BMs-DOX, and Tf-BMs-DOX in PBS (pH 7.4) were studied by dialysis. The result showed that DOX on BMs-DOX were released at a steady rate, without bursts. After dialyzing for 72 h in PBS (pH 7.4), the drug release rates of free DOX, BMs-DOX and Tf-BMs-DOX were 83.67%, 25.50% and 25.38%, respectively (Figure 1C). There was no significant difference in drug release rates between BMs-DOX and Tf-BMs-DOX. These results indicate that BMs-DOX and Tf-BMs-DOX are capable of sustained drug release, which could reduce their required frequency of administration and thus enhance their therapeutic effect.

### Detection of TfR expressed on cell surface

**Figure 2:**
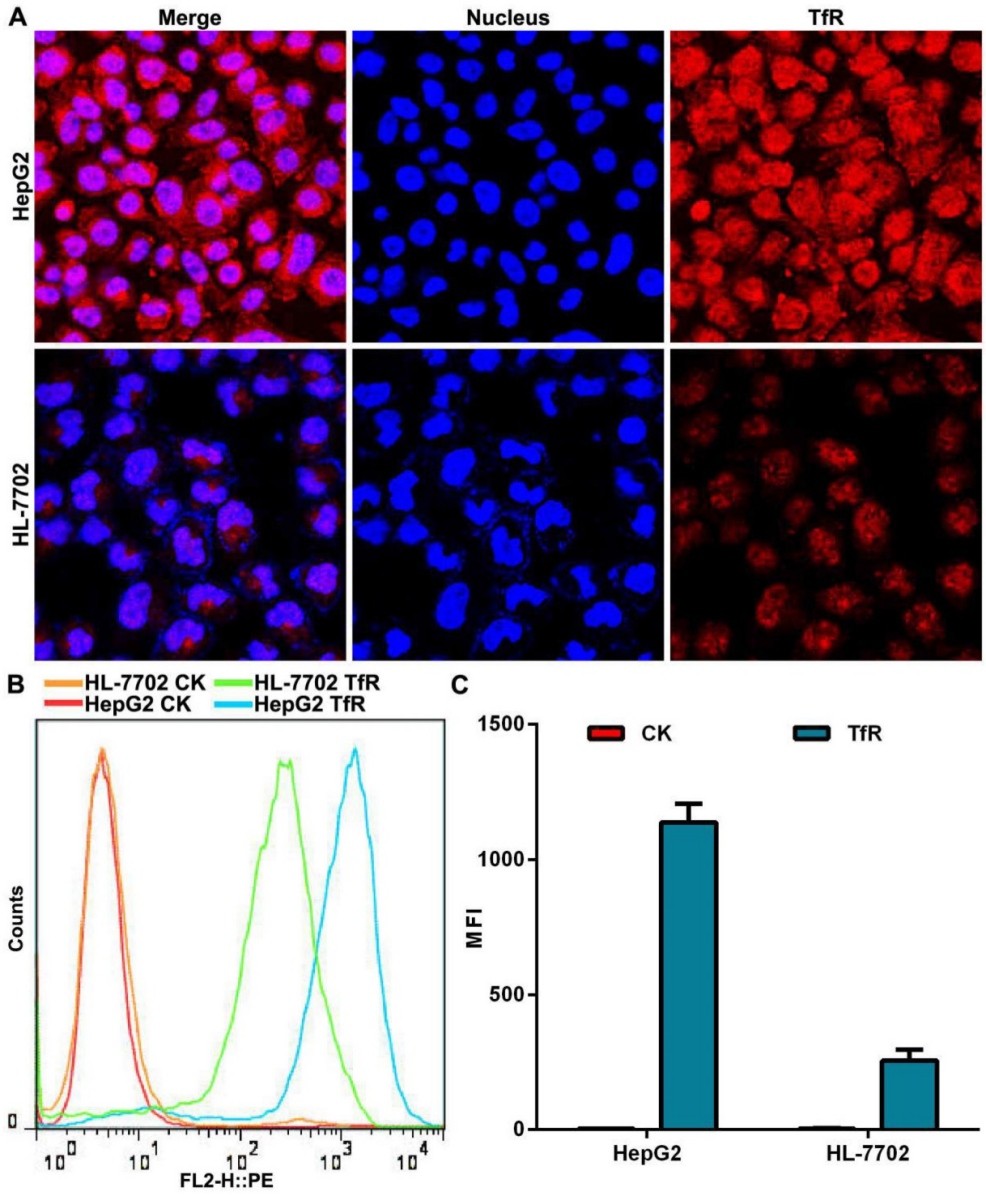
The expression of TfR on cell surface. (A) CLSM observation of TfR. Nuclei were stained with DAPI (blue fluorescence). Red fluorescence indicates TfR on cell surface. Scale bar = 50 µm. (B) Histogram of TfR detection was analyzed by flow cytometry. (C) Mean fluorescence intensity (MFI) of TfR detected by flow cytometry. Error bars: SD (n = 3).

We used a transferrin receptor antibody to analyze the expression of TfR on the surface of HepG2 cells and HL-7702 cells. Confocal laser scanning microscopy (CLSM) observation showed that the fluorescence intensity of TfR on the surface of HepG2 cells was stronger than that seen on HL-7702 cells (Figure 2A). Quantitative analysis of TfR expression by flow cytometry demonstrated that surface expression of TfR was 5.5 times higher on HepG2 cells than on HL-7702 cells (Figure 2B, C), which was consistent with our CLSM observation.

### Cellular uptake and intracellular localization of Tf-BMs-DOX

**Figure 3:**
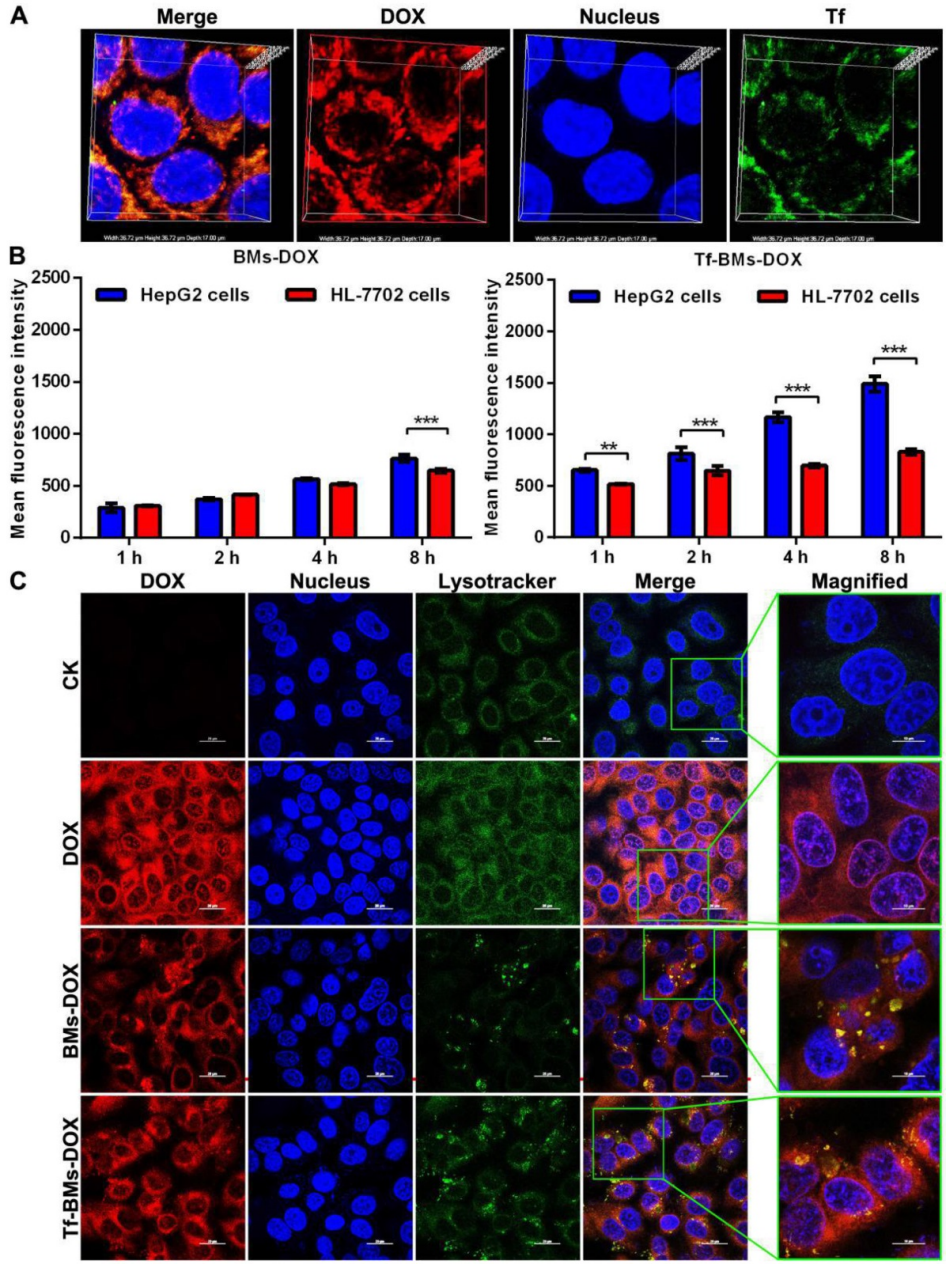
Cellular uptake and intracellular localization of Tf-BMs-DOX. (A) CLSM observation of HepG2 cells after incubation with 10 µg/mL for 1 h. (B) MFI of HepG2 and HL-7702 cells after incubation with 10 µg/mL BMs-DOX and Tf-BMs-DOX for different time. (C) Intracellular localization of Tf-BMs-DOX in HepG2 cells. Nuclei were stained with DAPI (blue fluorescence), red fluorescence represented DOX, and green fluorescence indicated Tf or lysosomes.

After incubation with Tf-BMs-DOX for 1 h, HepG2 cells were treated with Tf antibody (green fluorescence). CLSM imaging showed that the fluorescence of DOX overlapped with that of Tf (Figure 3A), indicating that both DOX and Tf were conjugated to BMs and that Tf-BMs-DOX can be internalized by HepG2 cells effectively.

To examine whether loaded BM uptake is Tf/TfR-dependent, we used fluorescence-activated cell sorting (FACS) to determine the quantity of DOX-loaded BMs taken up by HepG2 and HL-7702 cells after they were incubated with BMs-DOX and Tf-BMs-DOX for 1, 2, 4 and 8 h. We found that of the two cell types, HepG2 cells had a stronger capacity to take up Tf-BMs-DOX at all tested time points (Figure 3B). The difference in uptake between the cell types became more significant as the incubation time increased. In contrast, when cells were incubated with BMs-DOX, there was no difference in the uptake behavior between HepG2 and HL-7702 cells until the 8 h incubation timepoint (Figure 3B). These results showed that Tf-BMs-DOX have a stronger binding capacity to HepG2 cells compared with HL-7702 cells, demonstrating that Tf plays an important role in the recognition of HepG2.

To further explore the effect of the loading of Tf on the intracellular localization of DOX-loaded BMs, we also investigated the intracellular location of Tf-BMs-DOX in HepG2 cells (Figure 3C). After incubation with free DOX, BMs-DOX, and Tf-BMs-DOX respectively, HepG2 cells were treated with the lysosome-specific probe Lysotracker® Green DND-99. As shown in Figure 3C, the distribution of LysoTracker Green overlapped with the red fluorescence of DOX, indicating that DOX-loaded BMs are located within lysosomes in HepG2 cells. Furthermore, in cells treated with Tf-BMs-DOX, the fluorescence intensity was stronger than that of BMs-DOX treated cells, indicating that the loading of Tf enhance the binding and internalization of DOX-loaded BMs to cell surface.

### *In vitro* cytotoxicity assay of Tf-BMs-DOX

**Figure 4:**
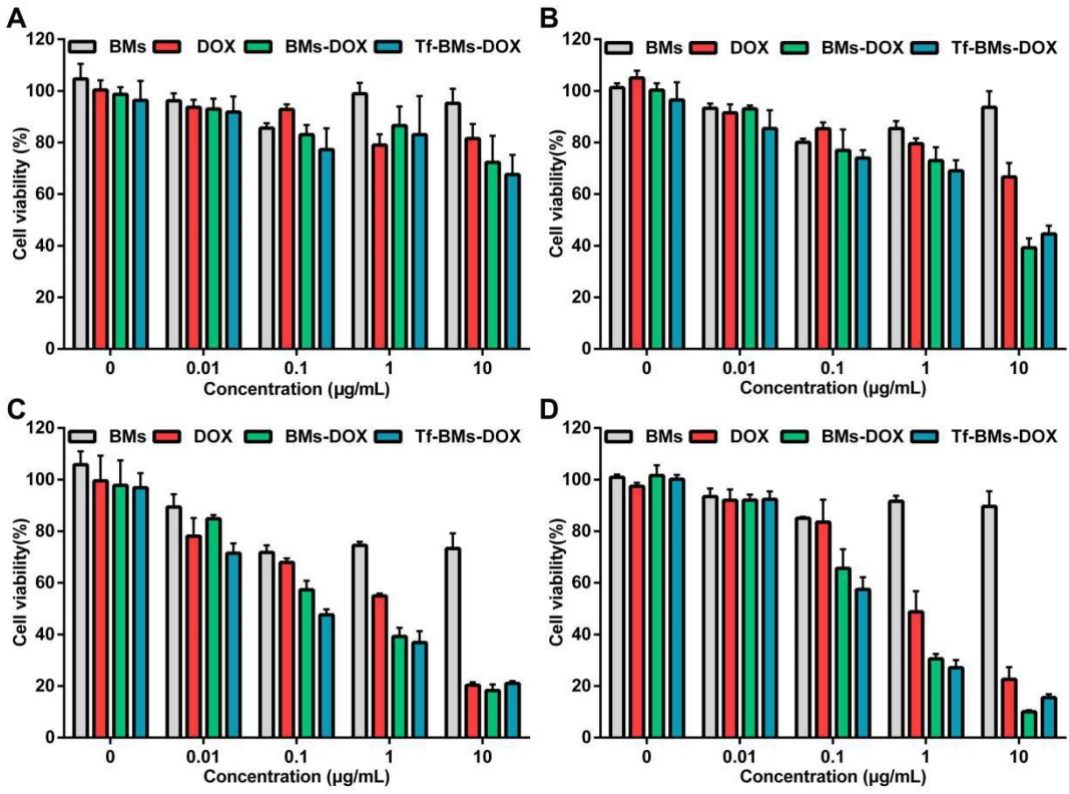
Cytotoxicity of BMs, DOX, BMs-DOX and Tf-BMs-DOX to HepG2 cells. Cell viability was evaluated by MTT assay after treatment with different regents for (A) 12 h, (B) 24 h, (C) 36 h, and (D) 48 h at the concentration of 0.01-10 µg/mL*.*

To evaluate the *in vitro* antitumor efficacy of DOX-loaded BMs, we used an MTT assay to assess cell viability (Figure 4). Cells were incubated with BMs, free DOX, BMs-DOX, and Tf-BMs-DOX at a concentration of 0.01-10 μg/mL and viability was measured at different time points (12-48 h) since the start of the incubation. The cytotoxicity of DOX, BMs-DOX, and Tf-BMs-DOX increased as the DOX concentration and the incubation time increased. Under identical conditions, BMs-DOX and Tf-BMs-DOX showed higher cytotoxicity than free DOX. When the DOX concentration was 0.1 μg/mL, the viability of the BMs-DOX, Tf-BMs-DOX, and DOX conditions was 65.57%, 57.48%, and 83.49%, respectively. At a higher concentration of 1 μg/mL, the viability of the BMs-DOX, Tf-BMs-DOX, and DOX conditions was 30.51%, 27.07%, and 48.74%, respectively.

**Figure 5:**
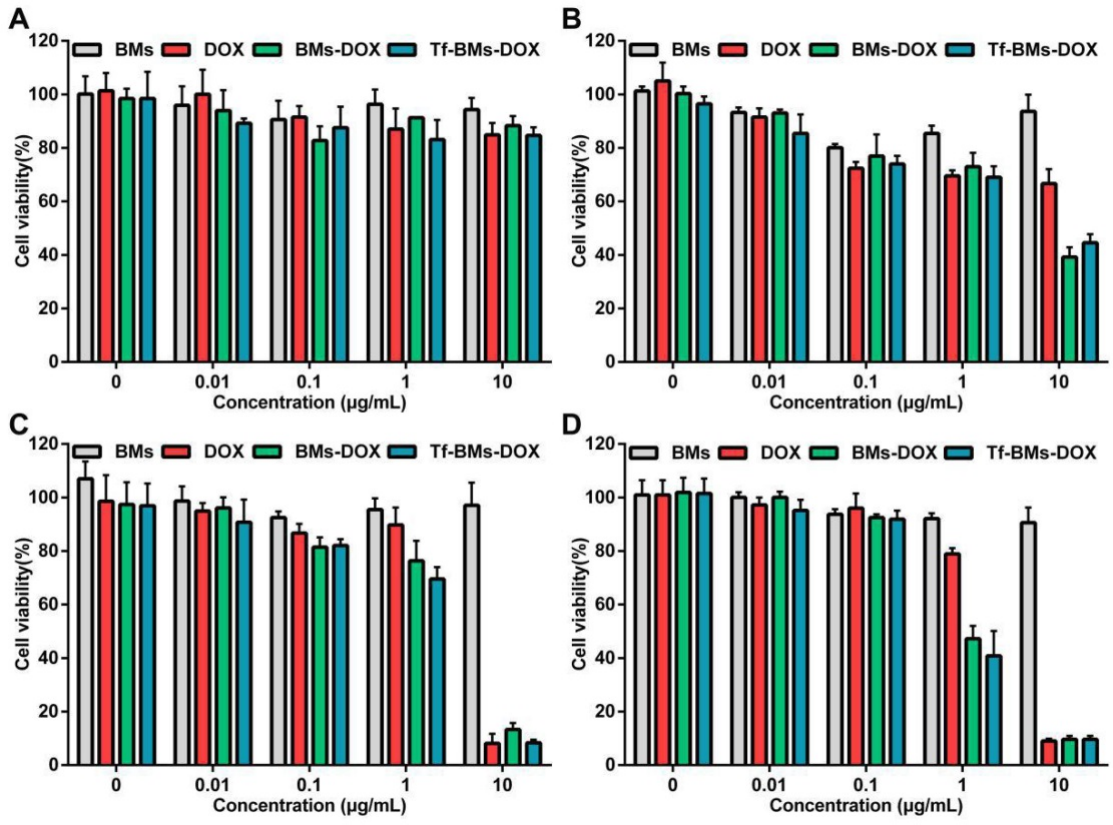
Cytotoxicity of BMs, DOX, BMs-DOX and Tf-BMs-DOX to HL-7702 cells. Cell viability was evaluated by MTT assay after treatment with different regents for (A) 12 h, (B) 24 h, (C) 36 h, and (D) 48 h at the concentration of 0.01-10 µg/mL.

The sensitivity of HL-7702 cells cytotoxicity mediated by DOX-loaded BMs was also evaluated using the same treatments as for HepG2 cells. As shown in Figure 5, the toxic effects of DOX, BMs-DOX, and Tf-BMs-DOX on HL-7702 cells became higher as the DOX concentration and incubation time increased, and the cytotoxicity of BMs-DOX and Tf-BMs-DOX was higher than that of free DOX. These results were consistent with those found for HepG2 cells. However, it was noticeable that BMs-DOX and Tf-BMs-DOX exhibited higher toxicity against HepG2 cells in comparison with HL-7702 cells under identical conditions. When HL-7702 cells were treated with 0.1 μg/mL BMs-DOX, Tf-BMs-DOX, or DOX for 48 h, the cell viability was 92.50%, 91.93%, and 96.00%, respectively, while the viability dropped to 47.27%, 40.82%, and 78.92% when the concentration was increased to 1 μg/mL. These viability values were overall higher for HL-7702 cells than for HepG2 cells. These findings suggest that BMs-DOX and Tf-BMs-DOX show lower cytotoxic effects on normal cells *in vitro*. In addition, non-loaded BMs exhibit no significant inhibitory effects on the growth of both HepG2 and HL-7702 cells at all tested concentrations and incubation times, demonstrating the safety and biocompatibility of BMs.

### Apoptosis assay

**Figure 6:**
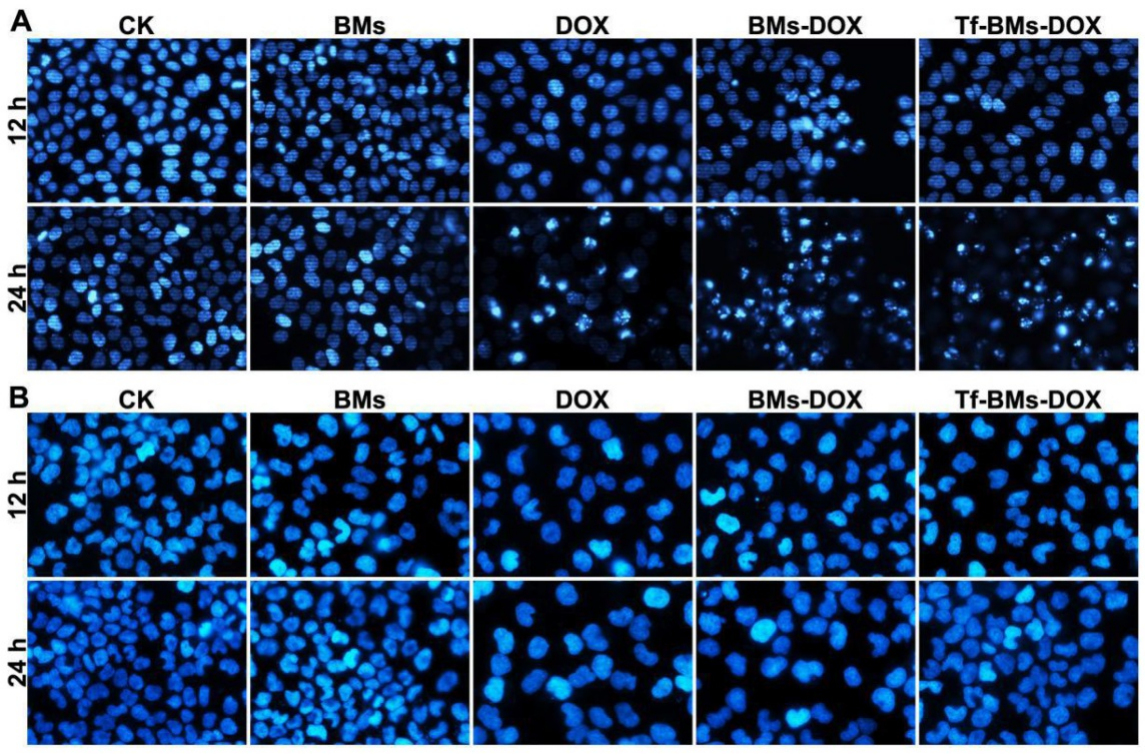
Apoptosis analysis of HepG2 and HL-7702 cells *in vitro*. Cells were treated with different regents at a DOX concentration of 0.5 µg/mL. For CLSM observation, cells were stained with Hoechst 33342. FACS was performed after Annexin V and PI double staining. CLSM images of (A) HepG2 and (B) HL-7702 cells after the treatment for 12 h and 24 h (400 ×).

**Figure 7:**
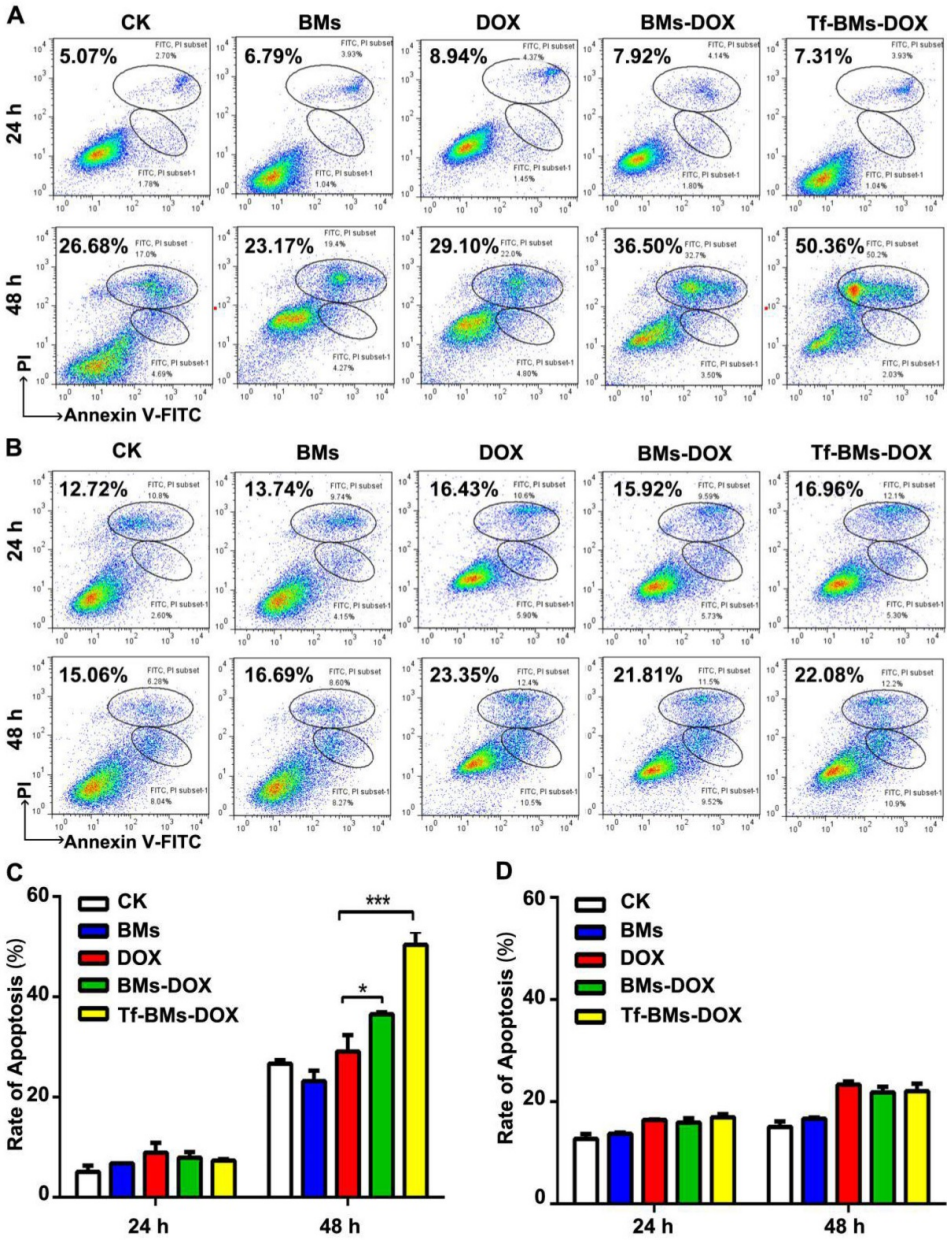
Apoptosis analysis of HepG2 and HL-7702 cells *in vitro*. (A) Flow cytometric analysis of Annexin V/PI double staining of HepG2 cells after the treatment for 24 h and 48 h. (B) Flow cytometric analysis of Annexin V/PI double staining of HL-7702 cells after the treatment for 24 h and 48 h. (C) The percentage of apoptotic cells of HepG2 cells after the treatment. (D) The percentage of apoptotic cells of HL-7702 cells after the treatment. *p < 0.05; **p < 0.01; ***p < 0.001. Error bar: SD (n = 3).

The apoptosis of cells after the treatment of Tf-BMs-DOX was studied using CLSM and flow cytometry, and morphological changes were observed after nuclei staining by Hoechst 33342. For HepG2 cells, DOX, BMs-DOX, or Tf-BMs-DOX treatment did not induce cellular apoptosis by 12 h. However, it did induced apoptosis after 24 h, as measured by chromatin condensation and increased nuclei fragmentation (Figure 6A). In contrast, no apoptosis was observed in HL-7702 cells after the treatment at the same DOX concentration for both 12 h and 24 h (Figure 6B). These results demonstrated that Tf-BMs-DOX induced apoptosis of HepG2 cells without apoptotic effects to HL-7702 cells after 24 h of treatment. Cellular apoptosis of HepG2 and HL-7702 cells was further quantitatively measured by flow cytometry (Figure 7A, B). FACS results revealed that there was no obvious apoptosis after all the treatments for 24 h in both cells. The percentage of apoptotic cells in HepG2 cells was 29.1%, 36.5% and 50.36% after treated with free DOX, BMs-DOX, and Tf-BMs-DOX, respectively, for 48 h (Figure 7C). However, the percentage of apoptotic cells in HL-7702 cells was 23.35%, 21.81% and 22.08%, respectively, indicating that Tf-BMs-DOX induce more severe apoptosis of HepG2 cells than free DOX and BMs-DOX. In addition, the results from both the CLSM and FACS assays demonstrated that non-loaded BMs exhibited no apoptotic effect on HepG2 cells, which was in accordance with the results of MTT assay.

**Figure 8:**
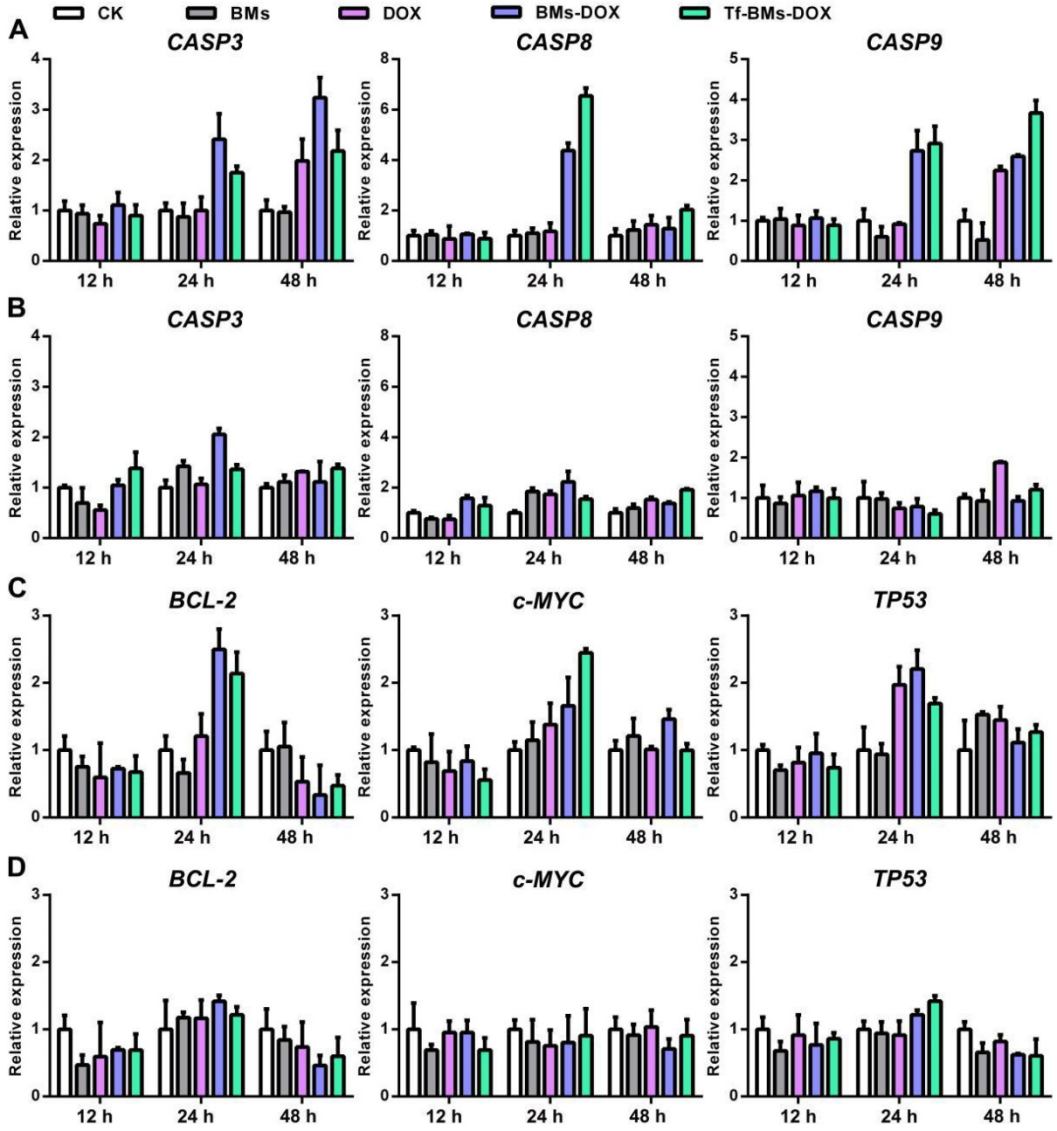
Gene expression in HepG2 and HL-7702 cells after the treatment of DOX-loaded BMs for 12 h, 24 h and 48 h. The expression of apoptosis-related genes *CASP*3, *CASP*8, and *CASP*9 in (A) HepG2 and (B) HL-7702 cells. The expression of tumor-related genes *BCL-*2, c-*MYC*, and *TP53* in (C) HepG2 and (D) HL-770 cells. Error bar: SD (n = 3).

It is well known that the primary mechanism of DOX toxicity is mediated through its ability to interfere with the duplication and transcription of DNA. To figure out the mechanism of Tf-BMs-DOX, we studied the expression of apoptosis-related genes, signaling pathway genes, and tumor-related genes within HepG2 and HL-7702 cells after the treatment with Tf-BMs-DOX (Figure 8). For both HepG2 and HL-7702 cells, the expression of *CASP3* was up-regulated after the treatment with DOX, BMs-DOX, or Tf-BMs-DOX for various times. The expression of *CASP9*, the initiator of the intrinsic apoptotic pathway, was up-regulated after the same treatment. The expression of *CASP8*, a key mediator of extrinsic apoptotic pathway, was up-regulated at 24 h and then down-regulated at 48 h (Figure 8A, B). In addition, we analyzed the expression of tumor-related genes *TP53*, *BCL*-*2*, and *c*-*MYC*. The expression of tumor-suppressing gene *TP53* was up-regulated after the treatment of DOX, BMs-DOX, or Tf-BMs-DOX for different amounts of time. This was also true for and *c*-*MYC*, which plays key regulatory roles in the cell proliferation, differentiation and apoptosis. In addition, the expression of *BCL*-*2*, an apoptosis inhibitory factor, was up-regulated after the treatment for 24 h and down-regulated after 48 h (Figure 8C, D). Of note, the expression of all these genes was more significantly changed in HepG2 cells compared with HL-7702 cells, which was consistent with the MTT assay and apoptosis analyses. These results suggested that the cytotoxicity and apoptosis caused by BMs-DOX and Tf-BMs-DOX resulted from the loading of DOX onto BMs.

### *In vivo* antitumor activity of Tf-BMs-DOX

**Figure 9:**
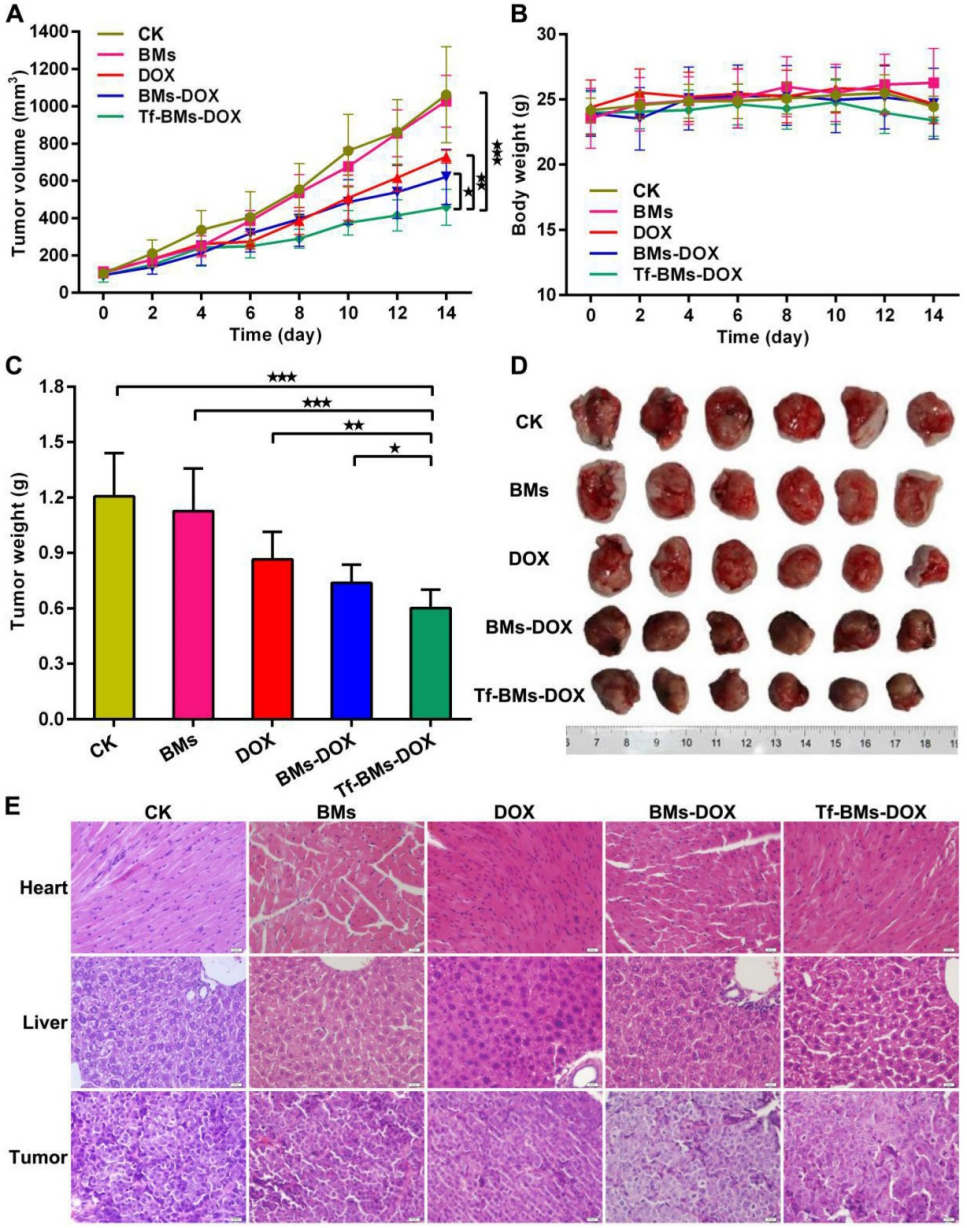
***In vivo* antitumor efficacy of DOX-loaded BMs. BABL/C nude mice were inoculated with HepG2 cells to establish xenografts models.** After average tumor volume reached 100 mm^3^, mice were treated with PBS, BMs, free DOX, BMs-DOX and Tf-BMs-DOX at the DOX dose of 4 mg/kg via tail vein injection. **(A)** Average tumor volume, **(B)** body weights and **(C)** average tumor weights of each group. **(D)** Photographs of tumor tissues. **(E)** H&E staining images of tumor, heart and liver tissues. *p < 0.05; **p < 0.01; ***p < 0.001 from Dunnett's multiple comparison test. Error bar: SD (n = 6).

We assessed the *in vivo* therapeutic effect of Tf-BMs-DOX was by monitoring HepG2 tumor growth in BALB/c nude mice xenografts models treated with various BMs (Figure 9). The tumor volume and body weight of each group were measured every 2 days. As shown in Figure 9A, treatment of mice with Tf-BMs-DOX inhibited tumor growth to 458.82 mm^3^, whereas the tumor burden was larger for the free DOX or BMs-DOX groups (729.78 mm^3^ or 620.74 mm^3^). The treatment of BMs showed no inhibitory effect on tumor growth (Figure 9A). The body weight of each group showed no significant difference throughout the whole treatment period (Figure 9B). The nude mice were sacrificed when the average tumor volume reached about 1000 mm^3^ in the control group, tumors were then harvested and photographed (Figure 9D). The average tumor weight in the Tf-BMs-DOX-treated group was 0.60 g, much smaller than that of the free DOX- or BMs-DOX-treated groups (0.87 g and 0.69 g, respectively) (Figure 9C). In addition to tumors, the heart and liver tissues of mice were collected for H&E staining. These tissues showed no obvious signs of pathological changes, including the typical cardiotoxicity of associated with DOX. Also, the histological sections of tumors revealed that there were fewer juvenile cells in the Tf-BMs-DOX treated group than in the free DOX or BMs-DOX treated groups, while necrotic area in the Tf-BMs-DOX group increased in comparison with free the DOX or BMs-DOX treated groups (Figure 9E). Altogether, these findings demonstrate that BMs may serve as excellent drug carrier, with enhanced therapeutic efficacy without unsafe side effects to major organs.

**Figure 10:**
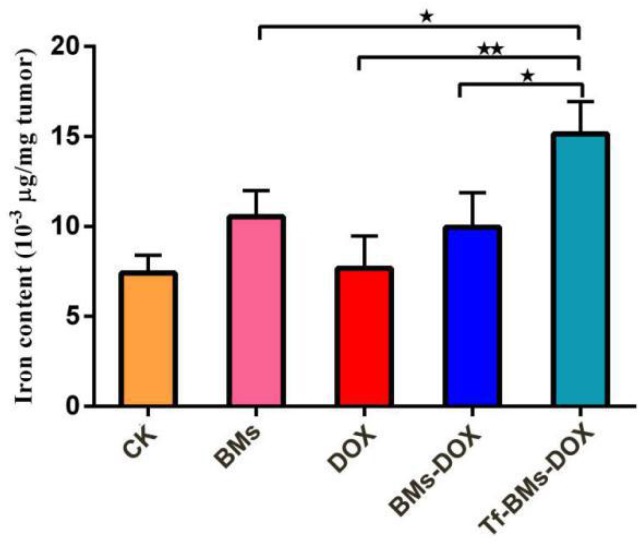
** Iron content of tumor tissue was detected by ICP-OES.** *p < 0.05; **p < 0.01; ***p < 0.001 from Dunnett's multiple comparison test. Error bar: SD (n = 3).

Finally, we used IPC-OES to determine the iron ions contents in tumor tissue. The result (Figure 10) revealed that the iron content (μg/mg tumor) in the group of Tf-BMs-DOX was detected as 15.14×10^-3^, which was higher than that of BMs-DOX group (9.95×10^-3^) and DOX group (7.68×10^-3^). Moreover, there was no significant difference between the BMs-DOX group and BMs group (10.54×10^-3^). Collectively, the results revealed that BMs can access to the tumor site and enter to the tumor cells through endocytosis.

## Discussion

In this study, we coupled Tf, a ligand of TfR, to BMs to study the targeting efficacy and cytotoxic effect of Tf-BMs-DOX on tumor cells. Compared to the original BMs-DOX, the active targeting ligand enhanced the ability to specifically target tumor cells, since the expression of TfR on the tumor cell surface is much higher than in normal cells. Thus, the drug-loaded BMs entered tumor cells more effectively, enhancing the inhibitory effect on tumor cell growth and survival, and reducing the toxic effects to normal cells.

We previously studied the inhibitory effect of BMs-DOX on tumor growth. H22 hepatoma tumor-bearing BALB/c nude mice were subcutaneously injected with 10 mg/kg BMs-DOX and free DOX adjacent to their tumors. These results showed that the inhibition rate of BMs-DOX was 86.8%, 8.2% higher than that of free DOX (78.6%). However, subcutaneous administration was only suitable for solid tumors that grew on the epidermis. For tumors residing within the body or metastatic cancer resulting from CTCs metastasized in the blood, subcutaneous administration was ineffective. In this study, we developed a potential tool to solve these problems through the creation of Tf-BMs-DOX, which targets specifically to TfR-expressing cells. 4 mg/kg (DOX content) Tf-BMs-DOX was administered following intravenous injection, which allowed this nanotherapeutic to act systemically, and subsequently recognize and bind to cancer cells with high expression of TfR. These results showed that the growth inhibition rate of Tf-BMs-DOX was 56.78%, 15.25% and 25.52% higher than that of BMs-DOX and DOX respectively. Compared with our previous work, the targeted drug-loaded BMs, constructed using a specific marker of tumor cells, enhanced the efficiency of tumor growth inhibition. Furthermore, this may show the potential for control of cancer metastasis and recurrence due to the active targeting capability of Tf-BMs-DOX.

BMs are a kind of natural magnetic nanoparticle, which have unique advantages in the field of cancer nanotherapeutics due to their unique characteristics. BMs, for example, can be used as a carrier of bioactive macromolecules, nucleic acids, antibodies, and antitumor drugs. However, their applications are limited due to the uncertainty of its biocompatibility and pharmacokinetics 49. In addition, the damage of the membrane of BMs during the process of purification and treatment is another obstacle to its more widespread use. Novakova et al. found that the structural integrity of BMs purified by 200 W ultrasound was more seriously damaged than that of BMs purified by 120 W 50, possibly due to the removal of functional proteins from BM membranes under high power. Qi et al. and Sun et al. revealed that extracted BMs showed good cytocompatibility without protease treatment 51, 52. BMs demonstrate biocompatibility, but show mild acute toxicity when the BMs concentration reaches LD=62.7 mg/kg 51. The toxicity of BMs may be due to their deposition in the body or non-human proteins on the membrane of BMs 26. However, highly pure and sterilized BMs exhibited no toxicity to mouse fibroblasts *in vitro* 53. As a result, BMs carry potential risk, but altogether are much less toxic than chemically synthesized nanoparticles.

## Conclusion

In this paper, we constructed bacterial nanoparticles loaded with DOX and transferrin (Tf-BMs-DOX) to study their anti-tumor effect. The expression of TfR on the surface of HepG2 cells was 5.5 times higher than that of HL-7702 cells, resulting in more efficient binding of Tf-BMs-DOX to HepG2 cells than HL-7702 cells. This resulted in Tf-BMs-DOX having a much higher ability to recognize HepG2 cells than BMs-DOX. *In vitro* experiments established that Tf-BMs-DOX enhanced tumor cell cytotoxicity and apoptosis through regulating the expression of tumor-related and apoptosis-related genes. Finally, after intravenous injection in HepG2 cell**-**bearing mice, Tf-BMs-DOX exhibited improved* in vivo* therapeutic efficacy compared to free DOX or BMs-DOX, without side effects to normal tissues. In summary, our findings provide a promising candidate for tumor treatment as well as a strategy for targeted nanomedicine.

## Figures and Tables

**Table 1:** Characterization of Tf-BMs-DOX, BMs-DOX and BMs

	Zeta potential (mV)	Hydrodynamic size (nm)	Drug loading (%)	Encapsulation efficiency (%)
BMs	-53.89 ± 0.98	418.02 ± 75.10	-	-
BMs-DOX	-40.65 ± 0.56	630.50 ± 29.90	48.22 ± 0.13	96.44 ± 0.25
Tf-BMs-DOX	-22.69 ± 0.53	702.86 ± 51.27	39.95 ± 0.31	79.90 ± 0.63
